# Supplementary material for: Proteomic Profiling Identifies Co-Regulated Expression of Splicing Factors as a Characteristic Feature of Intravenous Leiomyomatosis
Source: Cancers (Basel). 2022 Jun 13;14(12):2907. doi: 10.3390/cancers14122907 (PMC9221257; doi:10.3390/cancers14122907)
Supplement: Supplementary file 1 [file cancers-14-02907-s001.zip › supplementary figures.pdf]

Supplemental figures

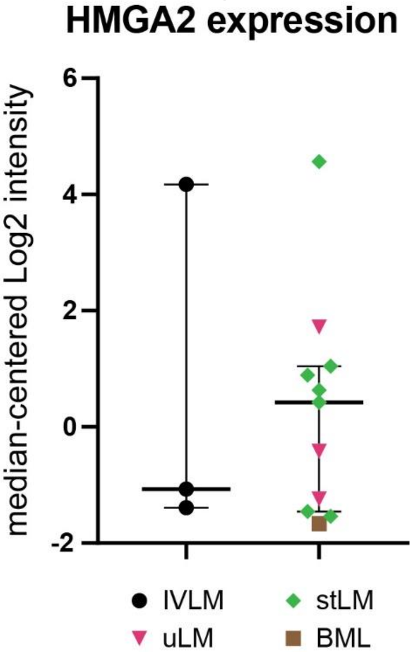

Figure S1: Expression levels of HGMA2 protein of each case in the cohort. The line and whiskers in plots represent median and interquartile range. Expression levels of this protein was not significantly different between IVLM and the other smooth muscle tumours in the cohort

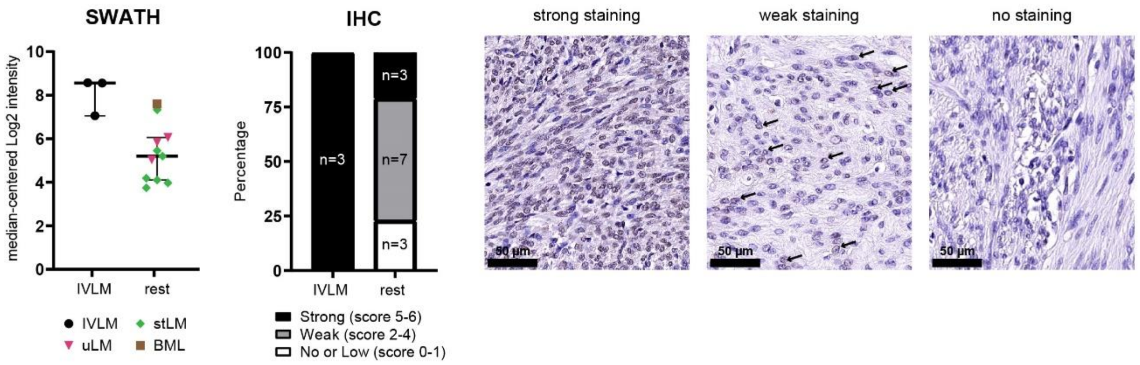

Figure S2: Comparative analysis of expression levels of SRSF3 protein by SWATH-MS with immunohistochemical staining (IHC) of the FFPE tissue sections from the same samples in the cohort. The line and whiskers in plots for SWATH-MS represent median and interquartile range. Stacked bar charts for IHC represents immunoreactivity of individual samples. Photomicrographs of representative samples with strong, weak and no staining for SRSF3 protein are shown. Scale bar represents 50 μm.
